# Comparison of postoperative pulmonary function and air leakage between pleural closure vs. mesh-cover for intersegmental plane in segmentectomy

**DOI:** 10.1186/1749-8090-6-61

**Published:** 2011-04-25

**Authors:** Kentaro Yoshimoto, Hiroaki Nomori, Takeshi Mori, Yasuomi Ohba, Kenji Shiraishi, Koei Ikeda

**Affiliations:** 1 Department of Thoracic Surgery, Faculty of Life Sciences, Kumamoto University, 1-1-1 Honjo, Kumamoto 860-8556, Japan; 2 Division of General Thoracic Surgery, Department of Surgery, School of Medicine, Keio University, Tokyo, Japan

## Abstract

**Background:**

To prevent postoperative air leakage after lung segmentectomy, we used two methods for the intersegmental plane: closing it by suturing the pleural edge (pleural closure), or opening it with coverage using polyglycolic acid mesh and fibrin glue (mesh-cover). The preserved forced expiratory volume in one second (FEV_1_) of each lobe and the postoperative air leakage were compared between the two groups.

**Methods:**

For 61 patients who underwent pleural closure and 36 patients who underwent mesh-cover, FEV_1 _of the lobe before and after segmentectomy was measured using lung-perfusion single-photon-emission computed tomography and CT (SPECT/CT). The groups' results were compared, revealing differences of the preserved FEV_1 _of the lobe for several segmentectomy procedures and postoperative duration of chest tube drainage.

**Results:**

Although left upper division segmentectomy showed higher preserved FEV_1 _of the lobe in the mesh-cover group than in the pleural closure one (*p *= 0.06), the other segmentectomy procedures showed no differences between the groups. The durations of postoperative chest drainage in the two groups (2.0 ± 2.5 vs. 2.3 ± 2.2 days) were not different.

**Conclusions:**

Mesh-cover preserved the pulmonary function of remaining segments better than the pleural closure method in left upper division segmentectomy, although no superiority was found in the other segmentectomy procedures. However, the data include no results obtained using a stapler, which cuts the segment without recognizing even the intersegmental plane and the intersegmental vein. Mesh-cover prevented postoperative air leakage as well as the pleural closure method did.

## Background

Advances in high-resolution CT scanning have led to frequent detection of peripheral T1N0M0 non-small cell lung cancers (NSCLCs). Although a randomized trial of lobectomy vs. limited resection for T1N0M0 NSCLC by the Lung Cancer Study Group in 1995 demonstrated that limited resection showed inferiority for prognosis and no advantage for postoperative pulmonary function compared to lobectomy [1], several studies conducted in Japan have demonstrated that segmentectomy is superior to lobectomy for preserving pulmonary function without worsening prognosis [2-7]. To preserve the pulmonary function of residual segments after segmentectomy, two techniques are considered important [8]: (1) sparing the intersegmental vein to preserve the venous drainage of residual segments, and (2) opening the intersegmental plane without closing it for sufficient re-expansion of the residual segments. However, opening the intersegmental plane causes postoperative air leakage. To prevent air leakage from the intersegmental plane, closing the pleural edge of preserved segments would be useful, but it would shrink the preserved segments, resulting in insufficient re-expansion. As another method to prevent air leakage, coverage of the opened intersegmental plane with polyglycolic acid (PGA) mesh and fibrin glue has been reported [9], but these materials are expensive. For that reason, comparison of the postoperative pulmonary function and duration of postoperative air leakage between the pleural closure and mesh-cover methods is important.

Since April 2005, the authors have conducted segmentectomy for c-T1N0M0 NSCLC, metastatic lung tumors and other lung nodules [10,11]. During the first term, April 2005 - December 2007, we closed the intersegmental plane by suturing the pleural edge of preserved segments to prevent postoperative air leakage. During the second term, January 2008 - March 2009, we opened the intersegmental plane with coverage by a PGA mesh and fibrin glue, not only to maintain re-expansion of the preserved segments but also to prevent air leakage. To evaluate the effectiveness of using PGA mesh and fibrin glue on the intersegmental plane for preserving pulmonary function and for preventing air leakage, we measured the preserved forced expiratory volume of lobes in one second (FEV_1_) using lung-perfusion single-photon-emission computed tomography and CT (SPECT/CT) and the postoperative duration of chest tube drainage. Subsequently, we compared data obtained from patients of the two groups.

## Methods

### Eligibility

	The Ethics Committees of Kumamoto University Hospital approved the study protocol for sublobar resection in patients with c-T1N0M0 NSCLC. Informed consent was obtained from all patients after a comprehensive discussion of the risks and benefits of the proposed procedures.

### Patients

Between April 2005 and March 2009, 198 patients with c-T1N0M0 NSCLC were treated with segmentectomy. Of the 198 patients, 166 patients underwent the conventional segmentectomy and 32 underwent the combined subsegmentectomy. Of the 166 patients who underwent conventional segmentectomy, 92 patients underwent both the pulmonary function test and lung-perfusion SPECT/CT before and after surgery. In addition to them, four patients with metastatic lung tumor and one with benign lung tumor were enrolled in the present study, constituting 97 patients in total.

#### Treatment for Intersegmental Plane

**Figure 1:**
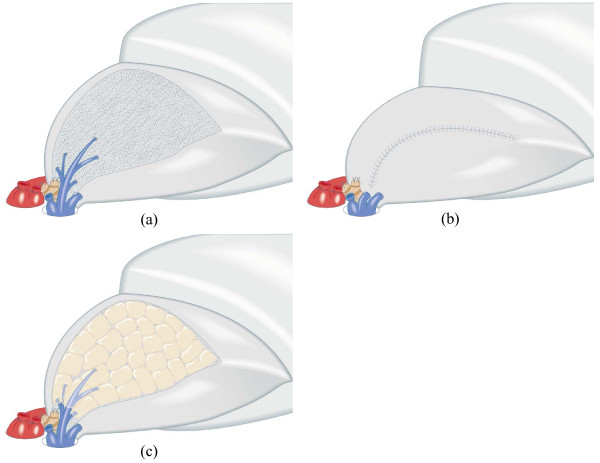
**Schema of pleural closure and mesh-cover treatment on intersegmental plane**. (a) Cross section of intersegmental plane preserving intersegmental vein. (b) The pleural closure method of intersegmental plane with continuous suturing of the pleural edge. (c) The mesh-cover method of intersegmental plane with coverage by polyglycolic acid mesh and fibrin glue.

During segmentectomy, the intersegmental plane was identified using the procedure reported by Tsubota et al. as follows [12]: (1) After the segmental bronchus was isolated, the whole lung was temporarily inflated; (2) The segmental bronchus was first ligated to retain the air inside the segment and then cut at the point proximal to the ligation; (3) Single-lung ventilation was restarted, thereby producing the inflated-deflated line between the resecting segments and preserving ones; and (4) The intersegmental plane was then dissected along the inflated-deflated line using electrocautery with the intersegmental vein as a guide, resulting in that the intersegmental veins were usually spared on the intersegmental plane enabling to preserve the venous drainage of adjacent segments (Figure 1a). To prevent postoperative air leakage, we treated the intersegmental plane using one of the following two methods. (1) During the first term of April 2005 - December 2007, the intersegmental plane was closed by continuous suturing the pleural edge of preserved segments (pleural closure) (Figure 1b). (2) During the second term of January 2008 - March 2009, the intersegmental plane was kept opened with coverage by PGA mesh and fibrin glue (mesh-cover) (Figure 1c). The pleural closure and mesh-cover groups respectively included 61 and 36 patients (Table 1).

**Table 1:** Patients' characteristics of the pleural closure and mesh-cover groups

	Pleural closure	Mesh-cover
Mean age (y.o.)	70 ± 9	68 ± 11
Male	29	12
Female	32	24
		
Pulmonary function		
VC (L)	3.0 ± 0.8	3.0 ± 0.8
%VC (%)	110 ± 14	112 ± 16
FEV_1 _(L)	2.1 ± 0.6	2.2 ± 0.6
FEV_1_/FVC (%)	73 ± 11	74 ± 8
		
Location of tumor		
Right upper lobe	10	9
Right lower lobe	16	9
Left upper lobe	25	14
Left lower lobe	10	4
		
Number of resected segments		
One segment	30	26
Two segments	27	9
More than 3 segments	4	1
		

Total number of patients	61	36

VC: vital capacity, FVC: forced vital capacity, FEV_1_: forced expiratory volume in one second

### Pulmonary Function Tests

Vital capacity (VC), forced vital capacity (FVC), and FEV_1 _were measured before and more than 6 months after surgery with a patient in a seated position using a dry rolling-seal spirometer (CHESTAC-9800DN; Chest Inc. Tokyo, Japan) according to American Thoracic Society standards [13].

#### Measurement of Pulmonary Function of Lobes

**Figure 2:**
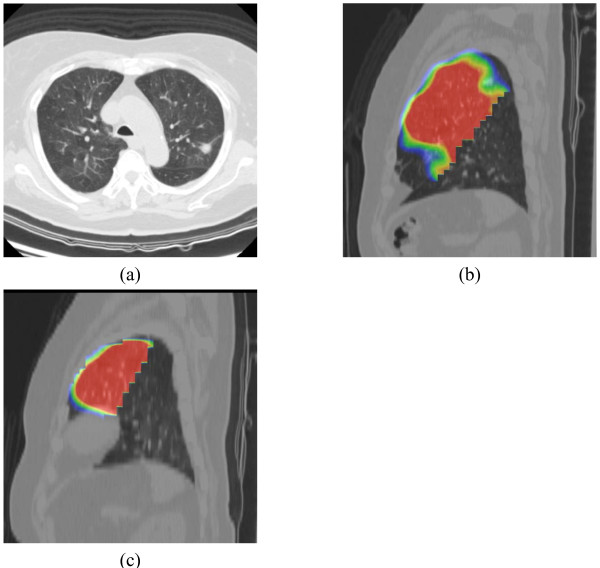
**Images of before and after segmentectomy**. (a) Axial image of CT before surgery, showing lung cancer in posterior apical segment of the left upper lobe. (b) Sagittal image of the lung-perfusion single-photon-emission computed tomography and CT (SPECT/CT) of the left upper lobe before operation. (c) Sagittal image of the lung-perfusion SPECT/CT of the remaining lingular segment after resection of upper division segmentectomy.

Lung-perfusion SPECT/CT was conducted both before and more than 6 months after surgery, at the same day with pulmonary function test. Preoperative and postoperative FEV_1 _of the lobe underwent segmentectomy was measured from pulmonary function test and lung-perfusion SPECT/CT, as previously reported [14-16]. Briefly, images of the lobe before segmentectomy and of the remained lobe after segmentectomy were traced on the CT image with a region of interest, of which the radioisotope (RI) was counted on the SPECT image (Figure 2).

The FEV_1 _of the lobe before (A) and after (B) segmentectomy was calculated from the preoperative or postoperative SPECT/CT according to the following formulae.

The percentage of preserved FEV_1 _of the lobe (C) was calculated according to the following formula:

### Resected sites compared between the two groups

	The percentage of preserved FEV_1 _of each lobe was compared between the pleural closure and mesh-cover groups in several resected sites of segmentectomy, i.e., resections of one segment of the right upper lobe, one segment of the left upper lobe, apical segment of the right lower lobe, apical segment of the left lower lobe, and the left upper division.

### Statistical Analysis

Student's *t*-test was used to compare the preoperative VC, %VC, FEV_1_, FEV_1_/FVC, preserved FEV_1 _of the lobe, percentage of preserved FEV_1 _of the lobe and the postoperative duration of chest tube drainage between the pleural closure and mesh-cover groups. Differences in mean percentage of preserved FEV_1 _of each lobe in each resected sites were analyzed by using multivariate analysis. Software (SPSS; SPSS Inc., Chicago, Illinois) was used for these analyses. Values of *p *< 0.05 were inferred as significant. All values in the text and table are given as mean ± SD.

## Results

No difference in preoperative pulmonary function was found between the pleural closure and mesh-cover groups, as shown in Table 1. In the pleural closure group, the respective mean values of FEV_1 _before and after surgery were 2.1 ± 0.6 and 1.9 ± 0.5 l, of which the mean percentage of postoperative FEV_1 _was 89 ± 9%. In the mesh-cover group, the respective mean values of FEV_1 _before and after surgery were 2.2 ± 0.6 and 2.0 ± 0.6 l, of which the mean percentage of postoperative FEV_1 _was 92 ± 8%. The mean percentage of postoperative FEV_1 _in the mesh-cover group was higher than that in the pleural closure group, with marginal significance (*p *= 0.09).

In the pleural closure group, the preoperative and postoperative FEV_1 _of each lobe that had undergone segmentectomy were 0.51 ± 0.20 and 0.22 ± 0.15 l, respectively, of which the mean percentage of preserved FEV_1 _of the lobe was 40 ± 20%. In the mesh-cover group, the preoperative and postoperative values were 0.52 ± 0.20 and 0.23 ± 0.12 l, respectively, of which the mean percentage of preserved FEV_1 _of the lobe was 46 ± 24%. The mean percentage of postoperative FEV_1 _of the lobe was not different between the two groups.

Table 2 presents the mean percentages of preserved FEV_1 _of the lobe in each resected site of the two groups. The pleural closure group showed a lower percentage of preserved FEV_1 _than the mesh-cover group for left upper division segmentectomy, with marginal significance (21 ± 10 vs. 35 ± 15%, *p *= 0.06). However, no significant difference in the values was found between the two groups at any other resected site, i.e., resections of one segment of the right or left upper lobe, or of an apical segment of the right or left lower lobe. The FEV_1 _values of the lobe before and after the upper division segmentectomy in the pleural closure group were 0.59 ± 0.21 and 0.13 ± 0.10 l, respectively, whereas the values in the mesh-cover group were, respectively, 0.47 ± 0.18 and 0.17 ± 0.10 l. Multivariate analysis of the mean percentages of preserved FEV_1 _of the lobe in each resected site of the two groups also showed no significant difference (*p *= 0.38).

**Table 2:** Mean percentage of preserved FEV_1 _of each lobe in each resected sites

	**Percentage of FEV**_**1 **_**of each lobe (%)**		
		
Resected site	Pleural closure	Mesh-cover	Difference
One segment of right upper lobe	38 ± 18 (n = 9)	35 ± 27 (n = 6)	p = 0.77
			
One segment of left upper lobe	46 ± 13 (n = 9)	52 ± 15 (n = 8)	p = 0.37
			
Apical segment of right lower lobe	59 ± 13 (n = 5)	63 ± 8 (n = 4)	p = 0.62
			
Apical segment of left lower lobe	46 ± 11 (n = 3)	44 ± 8 (n = 3)	p = 0.81
			
Upper division of left upper lobe	21 ± 10 (n = 10)	35 ± 15 (n = 4)	p = 0.06

Each Parenthesis shows number of the patients.

No significant difference was found in the respective durations of chest drainage, which were 2.0 ± 2.5 and 2.3 ± 2.2 days in the pleural closure and mesh-cover groups.

## Discussion

The results of this study elucidated the following points. (1) Mesh-cover is useful to preserve the pulmonary function of the residual lingular segment after the left upper division segmentectomy, although no difference was found between the mesh-cover and pleural closure methods at other resected sites. (2) Covering the intersegmental plane with PGA mesh and fibrin glue can prevent postoperative air leakage as well as the pleural closure.

The left upper division segment corresponds to the right upper lobe and has a larger lung volume than any other segment. Therefore, left upper division segmentectomy should be regarded as an exceptional procedure in segmentectomy for the preservation of pulmonary function. On the other hand, the left lingular segment corresponds to the right middle lobe and has smaller lung volume than the upper division segment, i.e. the upper division has six subsegments and the lingular segment has only four. This study showed that pleural closure in the upper division segmentectomy was associated with lower FEV_1 _of the remaining lingular segment more than the mesh-cover method, although no difference between the two methods was found at other segmentectomy sites. The following reasons might explain this outcome. (1) The remaining left lingular segment after left upper division segmentectomy has little lung volume, similar to the corresponding right middle lobe. (2) The functional volume of the lingular segment is likely to be decreased after left upper division segmentectomy because of the excessive upward bending and rotation of the lingular bronchus, similar to the occurrence of right middle lobe syndrome after right upper lobectomy [17]. (3) For these two reasons, pleural closure of the remained lingular segment shrink it and further decrease of the pulmonary function of the critically preserved lingular segment. The left upper division segmentectomy is a popular procedure for segmentectomy. Therefore, we must keep in mind that pleural closure in the left upper division segmentectomy preserves little pulmonary function of the remaining lingular segment. Furthermore, because the left upper division segmentectomy decreases the postoperative pulmonary function to a greater degree than segmentectomy of other kinds [12], left upper division segmentectomy should be examined separately in a controlled study of postoperative pulmonary function between the lobectomy and segmentectomy.

Recent development of stapling devices has added a new dimension to the technique for dissecting intersegmental plane. However, the present data include none related to closure of the intersegmental plane using a stapler. Although the pleural closure method in this study cut the lung tissue along the inflated-deflated line and spared intersegmental veins, the stapling method do not only cut the lung tissue without recognizing the intersegmental plane but also injure the intersegmental veins, which are instrumental for venous return of the residual segments. Therefore, segmental resection using a stapler will further decrease the pulmonary function of the remaining lobe, even compared to the pleural closure method described in this report. The use of staple devices in the dissection of intersegmental plane for preserving pulmonary function should be further evaluated in a separate study.

Results reported herein demonstrate that pleural closure does not decrease pulmonary function of the preserved segments compared to the mesh-cover procedure, except for left upper division segmentectomy. For left upper division segmentectomy, the intersegmental plane should be opened to preserve the pulmonary function of the residual lingular segment. Furthermore, results showed that coverage of the opened intersegmental plane using the PGA mesh and fibrin glue can prevent postoperative air leakage with the same degree of beneficial effect as pleural closure.

## Abbreviations

NSCLCs: non-small cell lung cancers; PGA: polyglycolic acid; FEV_1_: forced expiratory volume in 1 second; SPECT/CT: lung-perfusion single-photon-emission computed tomography and computed tomography; VC: Vital capacity; FVC: forced vital capacity; RI: radioisotope.

## Competing interests

The authors declare that they have no competing interests.

## Authors' contributions

This report reflects the opinion of the authors and does not represent the official position of any institution or sponsor. The contributions of each of the authors were as follows: KY was responsible for reviewing previous research, journal handsearching, drafting report. HN was responsible for quality checking and data processing. HN was responsible for project coordination. All authors have read and approved the final manuscript.
